# The importance and feasibility of hospital interventions to prevent and manage patient aggression and violence against physicians in China: a Delphi study

**DOI:** 10.1186/s12960-024-00914-z

**Published:** 2024-05-27

**Authors:** Yuhan Wu, Martina Buljac-Samardzic, Dahai Zhao, C. T. B. Ahaus

**Affiliations:** 1 Erasmus School of Health Policy and Management, Erasmus University Rotterdam, Rotterdam, The Netherlands; 2 School of International and Public Affairs, Shanghai Jiao Tong University, Shanghai, China

**Keywords:** Patient aggression and violence, Prevention and management, Hospital, Physicians, Interventions, Importance, Feasibility, Delphi study

## Abstract

**Background:**

Aggression and violence by patient (and their relatives/friends) is widely acknowledged as a serious occupational hazard, with physicians being particularly susceptible to witnessing and experiencing such incidents within hospitals. Research has shown that the negative consequences of such aggression and violence are not only felt at the individual level, but also at the team and organizational levels. Understanding how to prevent and manage this behavior towards physicians in hospitals is urgent and not fully researched. While there are many potentially effective interventions, it is unclear which ones would be valuable and feasible for Chinese hospitals. Because patient aggression and violence may occur more frequently in Chinese hospitals than in other countries, this suggests that cultural differences play a role and that tailored interventions may be needed.

**Method:**

We conducted a Delphi study to reach a consensus on the importance and feasibility of hospital interventions to prevent and manage patient (and their relatives/friends) aggression and violence against physicians in Chinese hospitals. Seventeen experts in China were invited to complete online questionnaires over three rounds.

**Results:**

After three rounds, consensus was achieved concerning 44 interventions, five other interventions were rejected, and no consensus was reached on another two. These interventions were clustered into eight categories: environment design, access and entrance, staffing and working practices, leadership and culture, training and education, support, during/after-the-event actions, and hospital policy. Each category is considered important in preventing and managing patient (and their relatives/friends) aggression and violence towards physicians in Chinese hospitals. This study also investigated the feasibility of the suggested interventions and found that 36 of the 44 interventions were considered not only relevant, but also feasible for implementation in Chinese hospitals.

**Conclusions:**

This study provides an overview of interventions that can be implemented in Chinese hospitals to prevent and manage patient (and their relatives/friends) aggression and violence before, during, and after a violent incident occurs.

## Introduction

Workplace violence in healthcare settings is recognized as a serious occupational hazard, and especially in hospitals [1, 2]. Many healthcare professionals worldwide experience verbal and physical violence at some point in their careers [3, 4]. Among healthcare professionals, physicians are particularly likely to witness and experience aggression and violence in the workplace [4, 5]. Although physicians encounter violence from different sources, patient and their relatives/friends have been identified as the most prevalent source of aggression and violence in hospitals [6, 7]. Consequently, this study focuses on patient (and their relatives/friends) aggressive and violent actions against physicians in hospitals.

The risk factors for the occurrence of aggression and violence are present at multiple levels, such as patient-related factors (e.g., under the influence of alcohol) physician-related factors (e.g., poor medical skills), and patient–physician interactions factors (e.g., poor physician–patient communication) [8]. Although inadequate services can also have negative impact on the patient [9], this study focuses on the multifaceted negative consequences for hospitals. At the individual level, it can have severe adverse effects on physicians’ psychology, emotions, work functioning (e.g., reduced job satisfaction, higher level of stress, and loss of confidence) [10–12] and even extend into their personal lives, resulting in an increasing need for family support and negative interactions with family members [6, 9]. Although the individual-level consequences have received most attention, this aggression and violence also affects behavior and performance at the team and organizational levels such as in influencing the team climate, lowering performance, increasing compensation costs, and reputational damage [13–15].

Given the detrimental impact of aggression and violence in healthcare settings, numerous studies have concentrated on preventing and managing workplace violence. The World Health Organization (WHO) proposed a guideline framework to prevent and manage workplace violence in healthcare that addressed preconditions, organizational interventions, environment interventions, individual-focused interventions, and after-the-event interventions [16]. The US Occupational Safety and Health Administration (OSHA) provided five guidelines for preventing workplace violence in healthcare that addressed: management commitment and worker participation, worksite analysis and hazard identification, hazard prevention and control, safety and health training, and recordkeeping and program evaluation [17]. Kumari et al. also suggested possible interventions to reduce workplace violence against physicians: at the individual level (e.g., training and communication skills); the organizational level (e.g., infrastructure changes and management policies); and the societal level (e.g., unbiased media reporting) in their review [18].

However, there is a lack of evidence on the effectiveness of these interventions. Therefore, Morpet et al. in a scoping review reviewed the effectiveness of interventions adopted by hospitals and identified risk assessment, staff education, and aggression management teams as evidence-based interventions that can reduce consumer-perpetrated violence [19]. Another systematic review categorized evidence-based interventions into three categories: pre-event preventive measures (e.g., violence prevention programs and risk assessment), interventions during the event (e.g., staying calm and applying de-escalation techniques), and post-incident measures (e.g., reflecting on incidents and organizational support) [1].

Given that the scope of workplace violence is broader than patient aggression and violence, including internal violence (violence from leaders/colleagues) and external violence (violence from patients/visitors) [20], it is sensible to place a particular emphasis on focused interventions for preventing and managing a specific source of violence against a specific type of healthcare professionals. The unique nature of patient (and their relatives/friends) aggression and violence against physicians necessitates tailored interventions to effectively address its challenges and negative effects.

Compared to European countries, physicians working in Asian countries experience more patient aggression and violence [21]. In the specific context of China, this problem has unique dimensions and challenges that require comprehensive investigation and addressing. Surveys conducted by the Chinese Hospital Management Society in 2005 revealed that the majority (over 73%) of healthcare staff in China were victims of such violence, including threats and taunts from patients and their relatives within hospital settings [21]. Furthermore, over the past few decades, the prevalence of patient aggression and violence against physicians has increased in China [22]. A recent systematic review conducted in China found that 62.4% of Chinese healthcare workers reported experiencing actual workplace violence, and particularly from patients [23].

The distinctive cultural, socioeconomic, and healthcare system factors in China underscore the need for a thorough examination of the importance and feasibility of hospital interventions tailored to the Chinese healthcare system. Although there are many suggested interventions, and some studies have examined the effectiveness of interventions elsewhere, it is not clear which are relevant and feasible in China given its cultural differences. Therefore, it is important to examine the importance and feasibility of interventions suggested in the literature in China.

As such, the main objective of this study is to reach a consensus regarding the importance and feasibility of hospital interventions to curtail and manage patient (and their relatives/friends) aggression and violence against physicians in Chinese hospitals. For these reasons, a Delphi study was conducted, aiming to contribute valuable insights and evidence-based recommendations that can enhance the safety and well-being of both patients and healthcare providers in China’s evolving healthcare landscape.

## Methodology

Based on the above analysis and given the scarcity and difficulty of experiment-based studies on interventions to prevent and manage patient (and their relatives/friends) aggression and violence [24, 25], this study opted to conduct a Delphi study. The Delphi method is mainly adopted when the existing knowledge is incomplete or subject to uncertainty and higher levels of evidence cannot be provided using other methods [26]. In this, we were aiming to reach a consensus among Chinese experts on the importance of hospital interventions, and to explore their feasibility to counter patient (and their relatives/friends) aggression and violence against physicians in Chinese hospitals. Three rounds were sufficient to reach consensus.

The panel of experts were recruited using authors’ own network and contained four types of participants: (1) management team members of Chinese hospitals and dedicated staff members (e.g., HR manager, quality and safety advisor) who hold the portfolio of patient aggression and violent behavior; (2) experts with experience in developing hospital policies on workplace violence (e.g., national/local health commission of China); (3) scientists who were specialized in patient–physician relationship (e.g., patient aggression and violence, patient–physician communication) in Chinese healthcare settings (scientists with a PhD degree and/or working experience > 10 years); (4) physicians who had experienced/witnessed patient (and their relatives/friends) aggression and violence in Chinese hospitals. We invited a maximum of two experts per region, hospital and research organization to ensure diversity of data sources. Since our aim was to derive hospital-level interventions, patients were not a target group.

The initial list of interventions (as presented in the first round of our Delphi study) was based on the results of our published systematic review about patient aggression and violence against physicians in hospitals, and that aimed to investigate the prevalence, risk factors, consequences, and prevention and management of patient (and their relatives/friends) aggression and violence against physicians in hospitals [8]. We started with an inventory of interventions mentioned in papers we had identified for our review plus additional papers found through a snowballing technique. Eventually, a list of 47 interventions were extracted from 32 related articles. Drawing on the WHO and OSHA guidelines [16, 17], we grouped the 47 measures into eight categories: (1) environment design, (2) access and entrance, (3) staffing and working practices, (4) leadership and culture, (5) training and education, (6) support, (7) during/after-the-event actions, and (8) hospital policy. All the interventions were translated from English into Chinese using the standard translation/back-translation technique by two researchers before each round of data collection [27].

The respondents completed online questionnaires during three Delphi rounds, where they rated each intervention as to ‘how important and how feasible is the intervention to prevent, cope, and/or manage patient (and their relatives/friends) aggression and/or violence against physicians in Chinese hospitals?’. More specifically, experts were asked to rate the importance and feasibility of each intervention relative to each other. A four-point scale was used (1 = not important to 4 = very important; and 1 = not feasible to 4 = very feasible). The first round took place in June 2023, the second in July 2023, and the third in August 2023. In each round, respondents were allowed three weeks to complete the questionnaire. After rating each intervention’s importance and feasibility, respondents had the opportunity to reformulate the intervention. At the end of each round, respondents also had the opportunity to add new interventions. In the second and third Delphi rounds, the list of interventions was based on the responses given in the previous round, including newly added, reformulated, and unchanged interventions that had scored somewhere between definite inclusion and exclusion (i.e., importance scores between 51 and 80%). The rules adopted for inclusion and exclusion of items were consistent with other Delphi studies [28–30].

Interventions that were rated as ‘very important’, or ‘important’ by at least 80% of the experts were immediately retained in the final list and those that were rated as ‘not important’, or only ‘moderately important’ by more than 50% the experts were excluded. New interventions, as well as interventions deemed important by 51% to 80% of the experts, were retained for re-evaluation in the next round of the Delphi study. This method, which includes feedback and the opportunity to reconsider initial answers, allowed the experts to reach consensus on all the interventions. In the third round, interventions that were not perceived as important by at least 80% of the experts were categorized as not achieving a consensus. Note that the exclusion and inclusion criteria in this study were based on the importance scores and not on the feasibility scores as it is not meaningful for hospitals to adopt feasible but unimportant interventions. However, our method can provide insight into the boundary implementation conditions for important but infeasible interventions.

## Results

Seventeen experts participated in all three rounds of this Delphi study, with no dropouts (response rate = 100%). Detailed information on the respondents is presented in Table 1.

**Table 1:** Background information on the panel of experts

Respondent	Job title	Gender	Educational background	Involvement in patient aggression and violence	Working years
R1	Physician	Male	Master	Witnessed	6–10 years
R2	Physician	Male	Master	Experienced	≤ 5 years
R3	Physician	Male	PhD	Experienced	16–20 years
R4	Physician	Withheld	Master	Witnessed	6–10 years
R5	Physician	Female	Master	Experienced	≤ 5 years
R6	Physician (head of department)	Female	Bachelor	Experienced	≥ 21 years
R7	Physician and security department manager in hospital	Male	Master	Experienced	≥ 21 years
R8	Physician and head of department of medical administration in hospital	Male	PhD	Witnessed	≥ 21 years
R9	Hospital HR manager	Female	Master	Witnessed	6–10 years
R10	Physician in patient-relations office staff	Female	Master	Witnessed	6–10 years
R11	Physician in patient-relations office staff	Female	Master	Policymaker in hospital	6–10 years
R12	Physician in patient-relations office staff	Female	PhD	Witnessed	16–20 years
R13	Head of department of medical safety in hospital and expert in related area	Female	Master	Scientist	≥ 21 years
R14	Expert in physician–patient communication	Female	PhD	Scientist/research	≥ 21 years
R15	Expert in physician–patient communication and health commission in China	Male	PhD	Scientist	11–15 years
R16	Health commission in China	Male	Master	Scientist/research	16–20 years
R17	Health commission in China	Female	Master	Witnessed and policymaker	16–20 years

Table 2 shows the flow of items through this Delphi study. During the three rounds, the panel added four new interventions to the list of 47 elements that we had gathered during the literature study. After three rounds, saturation was achieved with a final list including 44 items.

**Table 2:** Results three Delphi rounds

Response rate (*n* = 17)	Round 1 100%	Round 2 100%	Round 3 100%
Number of items	47	8	6
Included	37	4	3
Excluded	4	0	1
Reformulated	5	4	0
Unchanged	1	0	0
Newly suggested items	2	2	0

Unchanged means we used the same intervention in the next round

Table 3 shows the 44 interventions that made it through to the final list, together with their mean, level of agreement, SD, and assigned category. The interventions that were excluded or on which no consensus (NC) was reached are provided in Table 4. We discuss the level of importance in relationship to the level of feasibility of the included interventions, and particularly highlight differences in importance and feasibility.

**Table 3:** Round number, agreement, means and standard deviations of included elements

Round	Importance			Feasibility			Interventions	Category
	Agreement %	Mean	SD	Agreement %	Mean	SD		
1	100	3.65	0.49	100	3.65	0.49	Hospital security (e.g., 24-h coverage by security staff)	Environment design
1	100	3.53	0.51	100	3.53	0.51	Alarm systems (e.g., panic buttons, hand-held alarms) and reliable response system	
1	100	3.41	0.51	64.7	2.76	0.97	Separation of dangerous patients (e.g., psychiatric patients, drunk patients) from other patients	
3	100	3.35	0.49	82.4	3.06	0.66	Assigning security personnel (or dedicated coordinators) to intervene early in the event of loud noise resulting from conflicts	
1	100	3.18	0.39	100	3.41	0.51	Surveillance cameras with video recording	
1	94.1	3.18	0.53	64.7	2.88	0.78	Escape routes and safe rooms dedicated to physicians	
1	82.4	3.18	0.73	58.8	2.82	0.81	Protective measures in contact moments between physician and patient (and their relatives/friends)	
1	88.2	2.88	0.60	76.5	2.94	0.83	Electronic boards indicating approximate waiting times	
1	88.2	2.82	0.53	88.2	2.88	0.60	Adequate air conditioning (i.e., temperature /humidity/ventilation control) in waiting areas	
2	82.4	2.82	0.39	100	3.18	0.39	Relaxing and attractive colors in the hospital	
1	100	3.24	0.44	88.2	3.06	0.56	Security checks (e.g., metal detectors) at the hospital’s main entrance	Access and entrance
2	82.4	3.12	0.70	41.2	2.35	0.79	Risk assessment of patients’ aggression and violence based on their past behavior (e.g., history of violence, physically aggressive or threatening, verbal hostility)	
1	100	3.41	0.51	64.7	2.65	0.70	Adequate presence of staff at peak periods	Staffing and working practices
1	88.2	3.24	0.83	88.2	3.12	0.78	Gaining valid consent from patients (and their relatives, if necessary) before treatment	
1	94.1	3.41	0.62	88.2	3.24	0.66	Support from managers and hospital administration for physicians who experience patient (and their relative/friend) aggression and violence (e.g., paid leave and leadership concern)	Leadership and culture
1	82.4	3.00	0.61	92.1	3.18	0.53	Increasing leaders’ awareness of the impact of aggression and/or violence by patient (and their friends/relatives) on physicians’ well-being	
1	82.4	3.00	0.61	82.4	3.00	0.79	Involving physicians and patients in creating safety plans	
1	82.4	2.94	0.56	88.2	3.12	0.60	Leaders encouraging physicians (who have experienced/witnessed) to report patient (and their friends/relatives) aggression and/or violent incidents	
1	100	3.56	0.51	94.1	3.56	0.62	Training physicians in de-escalation techniques (e.g., showing understanding to patients)	Training and education
1	94.1	3.47	0.62	94.1	3.47	0.62	Training physicians in communication skills for use with patients and their relatives/friends	
1	100	3.41	0.51	100	3.35	0.59	Training physicians in managing and coping with aggressive and violent patients (and relatives/friends)	
1	94.1	3.35	0.61	76.5	3.24	0.83	Informing patients and their relatives/friends of the consequences of aggression and violence against physicians (in terms of legal, well-being, medical treatment)	
1	88.2	3.29	0.69	94.1	3.41	0.62	Informing physicians of their legal rights and available resources regarding encounters with patients’ (and by their relatives/friends) aggression and violence	
2	100	3.24	0.44	82.4	3.18	0.73	Professional coaching sessions for guidance on how to handle patient (and their relative/friend) aggression and violence	
1	88.2	3.18	0.64	94.1	3.24	0.56	Training to increase awareness among physicians on the importance of reporting every incident of patient (and their relative/friend) aggression and violence	
3	88.2	2.94	0.66	82.4	2.88	0.70	Training to improve the service attitude of healthcare providers towards patient (and their relatives/friends)	
1	82.4	2.94	0.75	70.6	2.88	0.70	Training physicians in self-defense	
1	100	3.65	0.49	100	3.53	0.51	A violence prevention program	Support
1	100	3.35	0.49	94.1	3.35	0.60	Offering peer support among physicians after patient (and their relative/friend) aggression and violence incidents	
1	94.1	3.35	0.56	88.2	3.24	0.66	Easy access to medical and mental health services for physicians	
1	100	3.24	0.44	88.2	3.12	0.60	Possibility for physician to request the presence of an additional health care worker during situations that the physician has identified as potentially dangerous	
1	82.4	3.12	0.70	88.2	3.29	0.69	Establishing conflict management teams in hospitals to avoid and respond to patient (and their relative/friend) aggression and violent incidents	
1	100	3.59	0.51	100	3.53	0.51	Providing legal aid to physicians	During/after-the-event actions
1	100	3.53	0.51	94.1	3.53	0.62	Providing immediate medical treatment to injured physicians	
1	94.1	3.24	0.56	88.2	3.18	0.64	Implementing formalized reporting procedures for patient (and their relative/friend) aggressive and violent incidents	
1	88.2	3.18	0.64	100	3.35	0.49	Providing information and support to the families of physicians after a patient (and their relative/friend) aggressive and violent incident	
1	88.2	3.00	0.71	88.2	3.00	0.71	24-h hotline for reporting patient (and their relatives/ friends) aggression and violence	
1	88.2	2.94	0.43	94.1	3.00	0.35	Comforting measures for patient (and their relatives/friends) (e.g., warm blankets and medication to reduce anxiety)	
1	100	3.82	0.39	82.4	3.29	0.92	Zero tolerance policy regarding patient (and their family/relatives) aggression and violence	Hospital policy
1	100	3.76	0.44	100	3.59	0.51	Weapon prohibition policy for patients or visitors	
1	94.1	3.41	0.80	100	3.41	0.51	Assigning a key senior management-level employee to handle patient (and their relatives/friends) aggressive and/or violent incidents	
3	100	3.35	0.49	100	3.35	0.49	Clarifying support staff’s responsibilities (e.g., healthcare guild and information desk, and registration and triage desk), standardizing the workflow and improving the work accuracy to avoid negative patient emotions	
1	94.1	3.25	0.56	94.1	3.24	0.56	Respecting patient privacy	
2	94.1	3.18	0.53	94.1	3.29	0.59	Clear hospital policy when incidents of aggression and violence by patient (and their relatives/friends) fall under civil law	

Agreement (importance) = the number of **important and very important** responses/the number of experts (*n* = 17)

Agreement (feasibility) = the number of **feasible and very feasible** responses/the number of experts (*n* = 17)

Mea*n* = sum of each expert’s ratings for each intervention/the number of experts (*n* = 17)

**Table 4:** Round number, agreement, means and standard deviations of excluded interventions

Round	Importance			Feasibility			Interventions	Category
	Agreement %	Mean	SD	Agreement %	Mean	SD		
1	47.1	2.35	0.86	47.1	2.41	0.80	One patient–one accompanying person policy	Access and entrance
1	35.3	2.29	0.92	64.7	2.76	0.83	Restricting visiting hours for patients’ relatives/friends	
1	29.4	2.18	0.64	17.6	1.82	0.73	Escorting physicians through different buildings in the hospital during nightshifts	Staffing and work practices
1	23.6	2.06	0.66	23.6	1.94	0.90	Escorting physicians to their transport home (car park, bus station) after nightshifts	
3	41.2	2.29	0.69	82.4	2.76	0.75	Implementing odor control in the hospital	Environment design
NC	64.8	2.71	0.77	94.1	3.06	0.66	Implement strict hygiene measures	
NC	64.8	2.65	0.86	88.2	2.94	0.83	Introducing pre-treatment nursing activities (e.g., medical guidance and taking blood pressure) for patients to reduce experienced waiting	

Agreement (importance) = the number of important and very important responses/the number of experts (*n* = 17)

Agreement (feasibility) = the number of feasible and very feasible responses/the number of experts (*n* = 17)

Mea*n* = Sum of each expert’s ratings for each intervention/the number of experts (*n* = 17)

*Environment design* There were seven suggested interventions in this category that were perceived as important and viable, referring to “hospital security”, “alarm system”, “assigning security personnel”, “surveillance cameras”, “adequate air conditioning” and “relaxing and attractive colors”. Four interventions (i.e., separation of dangerous patients from other patients, escape routes and safe rooms dedicated to physicians, protective measures in contact moments between physician and patient (and their relatives/friends), and electronic boards indicating approximate waiting times) were deemed important but less feasible. One expert suggested that interventions to separate dangerous patients from other patients, especially in emergency departments, is infeasible because it is difficult to identify potentially dangerous patients and execute separation measures without aggravating the patient. Although the intervention referring to applying odor control was rejected by the panel, one expert suggested that Chinese hospitals should increasingly prioritize enhancing the overall patient experience through environmental design, including plans for future improvements in waiting areas, dining spaces, and restroom facilities.

*Access and entrance* The interventions referring to security checks and risk assessment of patients were important, but the feasibility of the latter was questioned by most of the panel. In response to the feasibility of risk assessment, the experts had two points of concern. First, some experts commented that risk assessment was important, but that this intervention would require a complex linking of information between hospitals, and that this would currently be challenging to implement. Second, some experts expressed concerns that flagging patients based on a risk assessment might lead to patient stigmatization and the infringement of patient privacy, potentially exacerbating physician–patient conflicts and mistrust. This contradicted the view of some experts who believe that hospitals should construct blacklists based on risk assessments. Two interventions, referring to a ‘one patient–one accompanying person policy’ and ‘restricting visiting hours’, failed to achieve a consensus.

*Staffing and working practices* The interventions referring to gaining valid consent from patients (and their relatives, if necessary) before treatment and the adequate presence of staff at peak periods were important, but no consensus was reached on the feasibility of the latter. One expert explained that the number of physicians in hospitals was fixed, and the adequate presence of staff in this area might increase the workload of other physicians. Two interventions that referred to escorting physicians were rejected by the panel.

*Leadership and culture* All the interventions in this category were perceived as both important and feasible. Leadership plays a pivotal role in managing and coping with patient (and their relatives/friends) aggression and violence. Leaders can facilitate the establishment of an organizational safety climate by paying attention to the negative effects of aggression and violence for physicians, encouraging physicians to report violent incidents, and providing support to physicians who experience aggression and violence.

*Training and education* Most of the interventions related to training and education to prevent and manage patient (and their relatives/friends) aggression and violence were considered important and feasible. One expert suggested that training should encompass more than just managing and coping with aggressive and violent patients (and relatives/friends), and that identifying potentially aggressive and violent patients is also vital. Only two interventions (i.e., training physicians in self-defense, and informing patients and their relatives/friends of the consequences of their aggression and/or violence against physicians) did not achieve a full consensus on their feasibility. One expert commented that it is hard to inform patients and their relatives/friends at the hospital level because public education largely relies on government initiatives, social media campaigns, and other external channels. In addition, one expert commented that hospitals should recruit professionals or experienced physicians for training activities.

*Support* According to the panel, seeking support from both peers and organizations is not only important, but also feasible. One expert suggested that support from leaders is crucial since China is characterized by a high power-distance culture.

*During/after-the-event actions* All the identified interventions in this category were perceived as important and feasible.

*Hospital policy* All the related interventions were perceived as important and feasible by the panel. Two experts had the same comments on the ‘zero-tolerance policy regarding patient (and their family/relatives) aggression and/or violence’: that any zero-tolerance policy needs to be backed up at the national legal level, and is difficult for individual hospitals to implement.

## Discussion

The aim of this Delphi study was to explore the importance and feasibility of hospital interventions related to patient (and their relatives/friends) aggression and violence towards physicians in China. Consensus was reached on 44 interventions that were perceived as important for the prevention and management of patient (and their relatives/friends) aggression and violence against physicians in Chinese hospitals. These interventions were clustered into eight categories: environment design, access and entrance, staffing and working practices, leadership and culture, training and education, support, during/after-the-events actions, and hospital policy. Our findings indicated that all these intervention categories are important in preventing and managing patient aggression and violence. Saturation was reached after three rounds, as in the third round, the panel did not reformulate or put forward new interventions. There were only two interventions on which a consensus was not achieved. This study also investigated their feasibility and found that most of the important interventions were also considered feasible for implementation in Chinese hospitals.

In terms of environment design, respondents could consider two types of interventions: environmental factors and workplace design in hospitals. Among environment-related factors, our study found that air conditioning and color schemes (i.e., adequate air condition, and relaxing and attractive colors) in the hospital were considered both important and feasible. These supportive environmental factors have an influence not only on patient outcomes, but also on the satisfaction levels of both patients and physicians [31, 32], reducing the likelihood of patient aggression and violence. In terms of workplace design, hospitals should focus on security, alarm systems, reliable response systems, and surveillance cameras with video recording. These interventions also are widely reported elsewhere as part of a workplace violence prevention strategy in healthcare settings [33, 34]. The effectiveness of adopting surveillance cameras has also been considered in other studies [18, 19, 35]. More specifically, physical violence is decreased by the introduction of surveillance cameras and continuous monitoring of surveillance footage allows for the quick identification and rapid response to escalating behavior [35].

In terms of the interventions in the access and entrance category, security checks (e.g., metal detectors) at a hospital’s main entrance should be considered by Chinese hospitals since this intervention was perceived as important and feasible. This result is consistent with previous research which emphasizes security services at the main entrance and using weapon and metal detectors [16, 36]. However, whether patient risk assessments can be used in Chinese hospitals needs further consideration. Although our study deemed this intervention important, its feasibility was questioned by the experts. The same concerns are reflected in previous studies. Risk assessment advocates claim that risk assessments can be employed by hospitals to safeguard physicians and to reduce the incidence of violence [19, 35, 37]. However, its opponents are concerned that applying policies and procedures that flag individuals would lead to patient stigmatization and damage patient privacy [38, 39]. Although implementation of patient risk assessments is controversial, some countries have adopted practical measures to flag patient based on risk assessment. For example, the methods of flagging patient in some Canadian hospitals include a combination of symbols and colors (e.g., ‘purple dot’ sticker on patient charts) to indicate the risk [38]. However, such interventions are not straightforwardly translatable from one context to another since aggression and violence in healthcare settings is a culturally dependent concept [40]. Therefore, to enhance the feasibility of patient risk assessments in Chinese hospitals requires further research.

Leadership plays a pivotal role in preventing and managing patient aggression and violence in hospitals. Leaders should encourage physicians to report a violent incident as this has also been identified as an important and feasible intervention in other studies. More specifically, incident reporting is a key method for identifying trends in the causes of violence and factors for prevention [1, 41, 42]. Reported data can inform the development of appropriate and relevant prevention and response strategies for hospitals [42, 43]. Reflecting Chinese culture, which can be characterized as having a high power-distance [44], we found that support from managers and hospital administration is significant at the hospital level. This finding is in line with previous studies that emphasize the benefits of senior management support for safety programs in fostering hospital safety climates [45]. The Braverman seven-step workplace violence-prevention plan similarly stresses that getting support from the top is an essential step in workplace violence prevention [46].

In addition to support from leaders, this study showed that support from peers is also important and feasible, which is in line with other studies. Previous research has shown that implementing a peer support program for assaulted employees can lead to a reduction in the frequency of aggression and violence [47]. The buffering effect of support is significant when physicians experience patient aggression and violence [48]. Seeking peer support may provide the emotional support necessary to navigate challenging working conditions [14]. Further, having supportive and collaborative coworkers can foster motivation, increase job satisfaction, and enhance overall well-being in the workplace [49].

Moreover, providing training and education is seen as a key approach to preventing and managing patient aggression and violence in Chinese hospitals. Our study found that enhancing physicians’ skills including de-escalation techniques and communication skills, and in managing and coping with aggressive and violent patients (and relatives/friends) is important and feasible. These results are in line with other studies. In this regard, communication, de-escalation, and recognizing risky behaviors and triggers were identified as core elements to be addressed in training, and recognized as effective and person-centered mitigation strategies to reduce aggression and violence [1, 19]. Notably, our finding that self-defense techniques were not feasible has been similarly shown in other studies. Physicians have difficulty in applying self-defense techniques learned in training [50, 51] and there is no evidence that self-defense training reduces the incidence of violence in hospitals [19].

Furthermore, hospital policies for patient (and their relatives/friends) aggression and violence are also needed. A weapons prohibition policy for patients and visitors, and respecting patient’s privacy, were considered significant and viable methods for reducing patient aggression and violence, again a finding consistent with previous studies [16, 19]. Although having a zero-tolerance policy was perceived as important and feasible in our study, the effectiveness of this has been questioned in other studies. A major concern with a zero-tolerance policy is that it fails to discriminate between different causes of violence. This has resulted in employees in many healthcare settings not applying their ‘Refusal to Treat’ policy [19]. It has been recognized that zero-tolerance policies have not effectively reduced workplace violence among healthcare workers in Britain [52].

Importantly, the experiences of the experts in our study suggest a vital role for support staff within Chinese hospitals. Unlike patients in Western countries who often initially seek help from their general practitioner (GP) before they are admitted to a hospital, Chinese patients typically go directly to hospitals for treatment. This can lead to healthcare staff being overloaded in Chinese hospitals, especially in tertiary hospitals [53]. Consequently, patients without a GP referral and diagnosis have to rely heavily on support staff working on information and registration desks and on triage staff to guide them to the appropriate department for consultation and treatment. Mistakes made by support staff, such as directing patients to the wrong department, can easily trigger patient frustration and even violence towards physicians. Therefore, clarifying the responsibilities of support staff, standardizing workflows, and enhancing work accuracy to prevent such negative patient emotions and potential violence is considered an important intervention to avoid triggering violent patient behavior in China.

### Implications and limitations

Our study has practical implications for Chinese hospitals in terms of preventing and managing patient (and their relatives/friends) aggression and violence in different stages. To manage the period before violent events potentially occur, hospitals should provide professional training for physicians, especially in communication skills, skills on identifying potentially aggressive patients, and de-escalation approaches. Hospital policy should be established with the primary purpose of protecting the safety of physicians and clarifying when incidents of aggression and/or violence by patients (and their relatives/friends) fall under civil law. In addition, the design of the hospital environment, its access and entrance (e.g., security checks), and staff assignment should be considered in preventing and mitigating patient (and their relatives/ friends) aggression and violence before it takes place. During ongoing violent events, actions should focus on comforting measures for patient (and their relatives/friends), and de-escalation techniques. After such violent events, hospitals should provide support to physicians who have experienced aggression and violence in the workplace, in the form of leader support, peer support, and management support (e.g., representation and legal aid and medical support).

Our study has limitations that should be acknowledged. At first, since experts were recruited using authors’ own network, a selection bias might have occurred. The severity of the selection bias is limited as respondents were selected from a broad network of two authors and therefore include a diversity of participants from multiple hospitals, regions, and research organizations. In addition, it is crucial to note that patient aggression and violence are highly context-dependent phenomena. Therefore, interventions should take account of the specific national context, including the underlying risk factors associated with aggression and violence within Chinese hospitals. This contextual consideration is essential for ensuring the practical relevance and effectiveness of any interventions. Consequently, the generalizability of our findings to other cultures and contexts is limited. Nevertheless, this study can serve as starting point for other developing countries.

## Conclusions

This investigation, by conducting a three-round Delphi study, identified a broad consensus among experts on the importance and feasibility of hospital-based interventions for mitigating patient aggression and violence against physicians in China. In total, 44 interventions, later clustered in eight categories (i.e., environment design, access and entrance, staffing and working practices, leadership and culture, training and education, support, during/after-the-events actions, and hospital policy) were considered important. All the identified interventions that fall within the categories of leadership and culture, support, during/after-the-events actions, and hospital policy were deemed both important and feasible.

## Data Availability

The data collection scales and datasets created and/or analyzed through the present study are available from the corresponding author upon reasonable request.

## Abbreviations

WHO: The World Health Organization
OSHA: Occupational Safety and Health Administration
NC: No consensus
GP: General practitioner
